# Ursodeoxycholic Acid Modulates the Interaction of miR-21 and Farnesoid X Receptor and NF-κB Signaling

**DOI:** 10.3390/biomedicines12061236

**Published:** 2024-06-02

**Authors:** Chi-Yi Peng, Yi-Chun Liao, Yi-Chin Yang, Yi-Wen Hung, Lan-Ru Huang, Yen-Chun Peng

**Affiliations:** 1 Department of Veterinary Medicine, National Chung-Hsing University, Taichung 402202, Taiwan; pcynick@gmail.com; 2 Division of Gastroenterology, Department of Internal Medicine, Taichung Veterans General Hospital, Taichung 407219, Taiwan; s19001029@gmail.com; 3 School of Medicine, National Chung Hsing University, Taichung 402202, Taiwan; 4 Neurological Institute, Taichung Veterans General Hospital, Taichung 407219, Taiwan; jean1007@gmail.com; 5 Terry Fox Cancer Research Laboratory, Translational Medicine Research Center, China Medical University Hospital, Taichung 404327, Taiwan; hongiw@yahoo.com.tw; 6 Department of Medical Laboratory Science and Biotechnology, Central Taiwan University of Science and Technology, Taichung 40601, Taiwan; lrhuang@ctust.edu.tw; 7 School of Medicine, National Yang Ming Chiao Tung University, Taipei 11221, Taiwan; 8 Department of Post-Baccalaureate Medicine, College of Medicine, National Chung Hsing University, Taichung 402202, Taiwan

**Keywords:** farnesoid X receptor, lipopolysaccharide, microRNA-21, NF-κB signaling, ursodeoxycholic acid

## Abstract

(1) Background: This study investigates the effects of Ursodeoxycholic acid (UDCA) on NF-κB signaling, farnesoid X receptor (FXR) singling, and microRNA-21 in HepG2 cells. (2) Methods: HepG2 cells were treated with lipopolysaccharide (LPS) to simulate hepatic inflammation. The investigation focused on the expression of NF-κB activation, which was analyzed using Western blot, confocal microscopy, and Electrophoretic Mobility-shift Assays (EMSA). Additionally, NF-κB and farnesoid X receptor (FXR) singling expressions of micro-RNA-21, COX-2, TNF-α, IL-6, cyp7A1, and shp were assessed by RT-PCR. (3) Results: UDCA effectively downregulated LPS-induced expressions of NF-κB/65, p65 phosphorylation, and also downregulated FXR activity by Western blot. Confocal microscopy and EMSA results confirmed UDCA’s role in modulating NF-κB signaling. UDCA reduced the expressions of LPS-induced COX-2, TNF-α, and IL-6, which were related to NF-κB signaling. UDCA downregulated LPS-induced cyp7A1 gene expression and upregulated shp gene expression, demonstrating selective gene regulation via FXR. UDCA also significantly decreased micro-RNA 21 levels. (4) Conclusions: This study demonstrates UDCA’s potent anti-inflammatory effects on NF-κB and FXR signaling pathways, and thus its potential to modulate hepatic inflammation and carcinogenesis through interactions with NF-κB and FXR. The decrease in micro-RNA 21 expression further underscores its therapeutic potential.

## 1. Introduction

Ursodeoxycholic acid (UDCA) is a naturally secondary bile acid, which is transformed from chenodeoxycholic acid by gut microbiota. UDCA could be a key regulator of the intestinal barrier integrity, which is essential for lipid metabolism, and plays a protective role against inflammation and carcinogenesis [1,2,3,4]. Clinically, UDCA is also used as an established therapeutical drug, largely used for the dissolution of cholesterol gallstones, and the treatment of primary biliary cholangitis [3,5,6]. UDCA therapy can also effectively reduce serum levels of ALT and gamma-glutamyl transferase in patients with NASH [5,7,8,9]. Several studies have suggested that UDCA may have chemopreventive effects against inflammation and carcinogenesis [4,10]. UDCA could have an important role in hepatic and biliary carcinogenesis and may therefore be useful for the targeting treatment of chronic liver diseases and hepatic malignancies [4,11,12,13]. In terms of its potential as a chemo-preventive agent, UDCA has been shown to have anti-inflammatory and anti-oxidative effects [14,15,16,17]. In vivo and in vitro, UDCA has been shown to inhibit the proliferation of liver cancer cells, and this means its therapeutic potential for hepatic diseases [12,18]. Furthermore, UDCA is co-mutagenic and promotes cell transformation [19]. Nevertheless, it is still worth a better understanding of UDCA on chronic liver disease and the underlying mechanisms involved. Currently, the molecular mechanistic role that UDCA plays in the attenuation of hepatic carcinogenesis is not completely understood. NF-κB (nuclear factor κB) is a transcription factor that could upregulate several chemokines, and thus trigger several biological responses, including inflammation, immune responses, cell survival, and cell proliferation [20,21]. Dysregulation of NF-κB signaling has been linked to pathological response, including inflammation and carcinogenesis [22,23,24]. Moreover, NF-κB could contribute to cancer metastasis by regulating the expression of genes involved in cell migration and invasion [22,23]. NF-κB is an important regulatory transcription factor of carcinogenesis. Chronic inflammation persistently activates NF-κB signaling resulting in the promotion of carcinogenesis by creating a tumor microenvironment. In the hepato-biliary system, NF-κB orchestrates inflammatory crosstalk between hepatocytes and hematopoietic-derived cells resulting in the promotion of chemical hepatocarcinogenesis [25].

FXR (farnesoid X receptor) is a nuclear receptor that could regulate bile acid metabolism and homeostasis, with a particular role in the interaction of the liver–gut axis [26,27,28,29]. Dysregulation of FXR is associated with various liver diseases, including liver and biliary carcinogenesis [10,30,31]. Downregulation of FXR signaling has been associated with an increased risk of cancer in liver and biliary disease [31]. The relationship between FXR, SHP (Small Heterodimer Partner), and CYP7A1 (Cholesterol 7-alpha-hydroxylase) forms a critical regulatory pathway in the metabolism of cholesterol. FXR is activated by bile acids and subsequently induces the expression of SHP. SHP then acts as a transcriptional repressor, inhibiting the expression of CYP7A1, which is the rate-limiting enzyme in bile acid synthesis from cholesterol. This negative feedback loop helps maintain bile acid homeostasis and regulates cholesterol levels in the liver. Thus, we also check SHP and CYP721 to understand the intricate interplay between FXR, SHP, and CYP7A1.

MicroRNA is a small RNA molecule located in the cytoplasm and is involved in gene expression. It works with the RNA-induced silencing complex and this complex targets specific messenger RNA (mRNA), leading to mRNA degradation or inhibition of translation. Chronic liver diseases, such as non-alcoholic steatotic hepatitis, are associated with altered hepatic microRNA expression [32,33]. Consequently, miR-21 influences diverse cellular processes and has a role in hepatic carcinogenesis and inflammation. In liver pathological status, upregulated miR-21 exacerbates inflammation and fosters carcinogenesis, promoting cellular proliferation and impeding apoptosis, ultimately contributing to the development of liver cancer.

Lipopolysaccharide (LPS), a key component of the outer membrane of Gram-negative bacteria, plays a pivotal role in the initiation and perpetuation of inflammation. Studying LPS-induced responses in HepG2 cells is instrumental for unraveling the molecular mechanisms underlying hepatic inflammation and metabolism [34]. In this study, we aimed to elucidate the role of UDCA in liver inflammation and carcinogenesis, and therefore we investigated the mechanism by which UDCA interacts with NF-κB signaling, FXR, and miR-21 in HepG2 cells.

## 2. Materials and Methods

### 2.1. Cell Culture

A HepG2 hepatocellular carcinoma cell line was cultured in Dulbecco’s Modified Eagle’s Medium (Hyclone, Logan, UT, USA) supplemented with 10% fetal bovine serum (FBS) (Hyclone), 1% L-glutamine, and 1% penicillin/streptomycin (Sigma, St. Louis, MO, USA). When the cells reached 80–90% confluence, they were sub-cultured with 0.25% Trypsin–EDTA solution and resuspended in a fresh culture medium at an appropriate seeding density for continuous propagation. The experiments on HepG2 cells were performed when cells reached ~80% confluence.

### 2.2. Western Blot

The cell lysates from experimented cells were harvested and lysed in RIPA lysis buffer (Sigma St. Louis, MO, USA) containing protease inhibitors, 10 μM Phenylmethylsulfonyl fluoride (PMSF), and phosphatase inhibitors (Thermo, Waltham, MA, USA). The samples were incubated at 4 °C for 30 min, and then the supernatant was removed after centrifugation to obtain total protein content. BCA was used to determine the protein concentration. Aliquots (30 μg) of total proteins extracted from cultured cells were separated by electrophoresis through a 10% SDS–polyacrylamide gel at a constant voltage of 70 V for 30 min, followed by 110 V for 90 min in running buffer (25 mM Tris–HCl pH 8.3, 192 mM glycine, and 0.1% SDS). The proteins were subsequently transferred onto a nitrocellulose membrane at 350 mA in transfer buffer (25 mM Tris–HCl pH 8.3, 150 mM glycine, and 5% *v*/*v* methanol) for 90 min. The nitrocellulose membrane was then blocked with 5% BSA in Tris-buffered saline (TBS) (20 mM Tris pH 7.6, 137 mM NaCl) for 1 h. Immunostaining was performed using NR1H4 antibody (1:1000 dilution), NF-κB p65 antibody (1:1000 dilution), Phospho-NF-κB p65 (Ser536) antibody (1:1000 dilution) (Cell Signaling Technologies, Danvers, MA, USA), and GAPDH antibody (1:2000 dilution). Subsequently, the membranes were collected, washed, and incubated with appropriate HRP-conjugated secondary antibodies at room temperature for approximately 1 h and a secondary polyclonal mouse anti-goat antibody HRP conjugate (1:5000) (Cell Signaling, Technologies, Danvers, MA, USA). Blots were developed using HRP and Trident Femto Western HRP Substrate (GeneTex, Irvine, CA, USA). Data were captured using a Geliance CCD camera (Perkin Elmer, Waltham, MA, USA), and analyzed using ImageJ software (version 1.54). The optical densities of the bands were quantified using ImagePro plus 5.1 (Media Cybernetics, San Diego, CA, USA), and the relative amounts of each protein were quantified as the ratio of the protein quantity to that of GAPDH indicated underneath each gel.

### 2.3. Quantitative PCR

Total RNA was extracted from HepG2 cells (1 × 10^6^ cells/mL) and then incubated with LPS (1 μg/mL) alone or in the presence of UDCA (500 μM) or the corresponding vehicle for 24 h. Total RNA was extracted from cell cultures using Trizol reagent (Bio-Rad Laboratories, Hercules, CA, USA), followed by genomic DNA removal with DNase digestion using a Turbo DNA-free kit (Applied Biosystems, Foster City, CA, USA). Subsequently, a purified 500 ng of total RNA was reverse transcribed with Oligo (dT) by utilizing the Superscript III First-strand Synthesis SuperMix (Invitrogen, Carlsbad, CA, USA) to create cDNA templates. Then, the qPCR analysis was performed with QuantStudio 3 Real-time PCR system (Thermo Fisher, Waltham, MA, USA). The value of the target gene was normalized to that of GAPDH. The relative standard curve method (2^−ΔΔCt^) was used to determine relative mRNA expression, with GAPDH as an internal reference for normalization. The forward and reverse sequences of the qPCR primers, which were created and synthesized, are displayed in Table 1.

### 2.4. ELISA

The IL-6 concentrations were measured using a commercially available ELISA kit (R&D Systems # D6050B), according to the manufacturer’s instructions. Briefly, samples and standards were added to a 96-well plate pre-coated with an anti-IL-6 antibody and incubated at room temperature for two hours. After washing, a biotinylated secondary antibody specific for IL-6 was added and the plate was incubated for an additional hour. Following another series of washes, a streptavidin–HRP conjugate was added and incubated for 30 min. The plate was then washed again and a substrate solution was added to develop color. The reaction was stopped with a stop solution, and the absorbance was measured at 450 nm using a microplate reader. The IL-6 concentrations in the samples were calculated from a standard curve generated using known concentrations of recombinant IL-6. This method allowed for the precise quantification of IL-6, enabling the assessment of UDCA’s anti-inflammatory effects in the context of NF-κB signaling.

### 2.5. Quantitative Analysis of MicroRNA-21 Levels

To evaluate the effect of UDCA on micro-RNA 21 in LPS-induced HepG2 cells, the expression of microRNA-21 levels in each sample was assessed using SYBR green-based relative quantification assay and microRNA-21-specific oligonucleotide sequences (forward primer: 5′-CGGGATCCTGGGGTTCGATCTTAACAGGC-3′ and reverse primer: 5′-CGGAATTCCCACAATGCAGCTTAGTTTTCC-3′) were determined using Real-time quantitative PCR (RT-qPCR, ABI 7500, Applied Biosystems). U6 SnRNA (forward primer: 5′-CGCTTCGGCAGCAGCACATATACTA-3′ and reverse primer: 5′-CGCTTCACGAATTTGCGTGTCA-3′) was used as an endogenous control for evaluating the expression levels of microRNA-21 intreated cells relative to controls. The reaction conditions followed for RT-qPCR analysis were as follows: an initial step of denaturation at 95 °C for 2 min, followed by 40 cycles of denaturation for 30 s at 94 °C, annealing for 30 s at 58 °C and extension for 30 s at 72 °C. A melt curve analysis was also performed at the end of all of the cycles for 10 min to differentiate between true amplicons and primer dimers in each sample. Each reaction was performed in triplicates, and mean values were used for calculating the relative fold change.

### 2.6. Confocal Microscopy

Confocal fluorescence microscopy was performed for the evaluation of NF-κB p65 expression, nuclear translocation, and phosphorylation. HepG2 cells were cultured and treated as per experimental conditions. Following appropriate treatments, the cells were fixed with 4% paraformaldehyde for 10 min, a standard technique for preserving cellular structures. Subsequently, the cells were permeabilized with 0.5% Triton × 100/PBS for 8 min, which allowed antibodies to penetrate the cell membrane and interact with intracellular targets. Immunofluorescent staining was performed to specifically label NF-κB within the cells. This involved incubating the cells with anti-phospho-NF–κB p65 (1:200, Cell Signaling) overnight at 4 °C, followed by the addition of Alexa 488-conjugated secondary antibodies at a concentration of 1:500 for 1 h at room temperature. This step ensures precise detection of the target protein. Additionally, Hoechst 33342 (Invitrogen) at 1 μg/mL was used for nuclear staining to visualize the cell nuclei, aiding in the localization of NF-κB within the cellular context. The images were captured with a Leica confocal microscope (Leica Microsystems, Wetzlar, Germany).

### 2.7. Electrophoretic Mobility-Shift Assays (EMSA)

HepG2 cells were seeded at a density of 1 × 10^6^ cells/well in 6-well plates and treated with LPS (1 μg/mL) in the presence or absence of UDCA (500 μM) for 24 h. Nuclear extracts from cells were prepared with a nuclear extraction kit (Abcam, Cambridge, England, UK) according to the manufacturer’s instructions. Electrophoretic mobility shift assay was performed using an EMSA Assay Kit (GS-0030, Signosis, Inc., Santa Clara, CA, USA) and oligonucleotide probe sets specific for NF-κB (Panomics, Redwood City, CA, USA) according to the manufacturer’s instructions. For the assay, 5 μg of nuclear protein was incubated with 1 μg poly (dI-dC), 2 μg 5X binding buffer, and 0.2 μg of NF-κB transcription factor probe in a microcentrifuge tube at 22 °C for 30 min in a PCR machine. Following this, the binding reaction mixture was loaded onto 6.5% non-denaturing polyacrylamide gels, and electrophoresis was carried out to separate the NF-κB DNA complex from the free DNA probe. The resulting membrane was then visualized using a chemiluminescence system.

### 2.8. Statistical Analyses

Each group in this study (*n* = 3) was collected in each experiment. Results were presented as mean ± standard deviation. PCR results presented in box plots were performed 5 times (*n* = 3). ELISA was also presented by box plots (*n* = 4). All experiments were repeated independently at least 5 times. The single-factor analysis of the Kruskal–Wallis test was used to evaluate differences between groups. After the Kruskal–Wallis test analysis, statistical significance was set at *p* < 0.001.

## 3. Results

### 3.1. UDCA Attenuated LPS Activated NF-κB Expression

**Figure 1:**
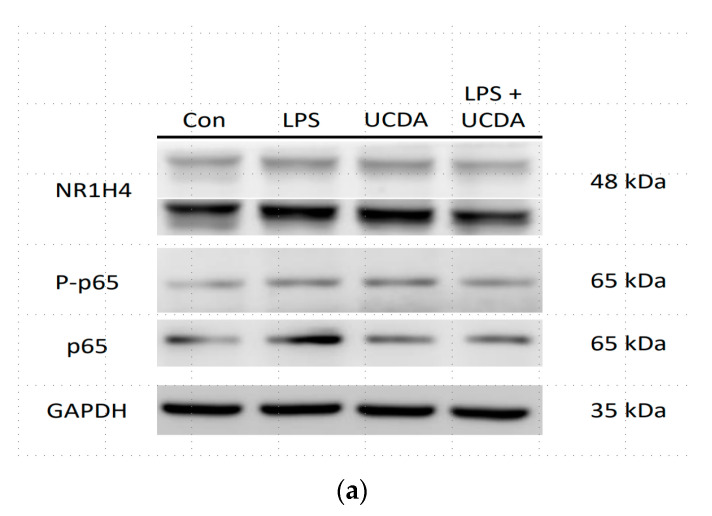

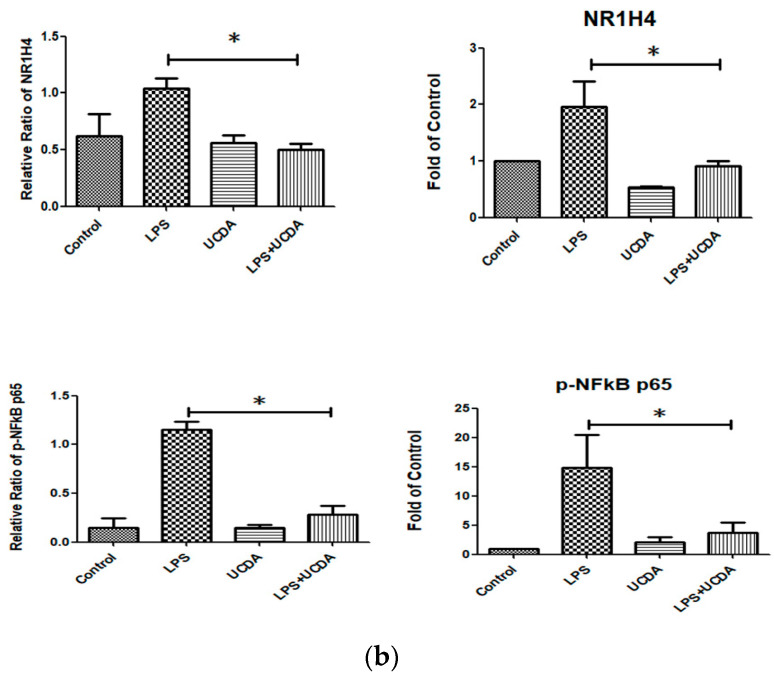
UDCA attenuates LPS-activated NF-κB expression. Expression of NF-κB p65, phospho-FN-κB p65, and farnesoid X receptors (NR1H4) (**a**) and the optical densities of the bands were quantified (**b**). HepG2 cells were treated with LPS in the presence and absence of UDCA. Control cells (Con) and UDCA alone determine the UDCA effect on LPS in HepG2 cells. The inflammation response of HepG2 cells was induced with the LPS (1 μg/mL) and under the UDCA (500 μM) for 24 h. Lysates were subjected to immunoblotting using anti-NF-κB/p65, anti-phospho-NF–κB/p65 antibodies. Protein expression was studied by Western blotting and GAPDH served as the control. * *p* < 0.001 Kruskal–Wallis test.

**Figure 2:**
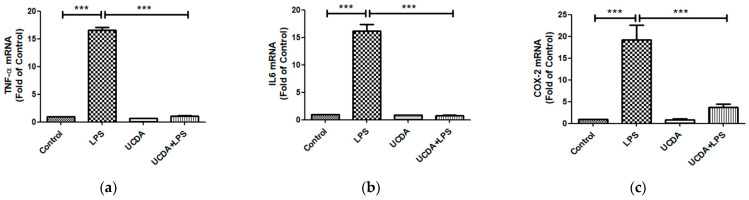

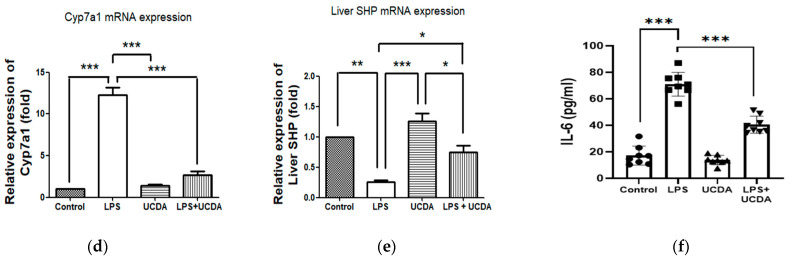
UDCA regulates LPS-induced FN-κB and FXR target genes and proteins. UDCA regulates LPS-induced NF-κB target production of TNF-α, IL-6, and COX-2, and FXR target genes mRNA in HepG2 cells. HepG2 cells (1 × 10^6^ cells/mL) were incubated with LPS (1 μg/mL) alone or in the presence of UDCA (500 μM) or the corresponding vehicle for 24 h. LPS could upregulate expression of TNFα (**a**), IL-6 (**b**), and COX-2 (**c**), which could be downregulated by co-treatment with UDCA. Glyceraldehyde 3-phosphate dehydrogenase (GAPDH) was used as an internal control to normalize the target gene’s expression. UDCA decreases LPD-induced liver cyp7a1 mRNA expression (**d**) and increases liver shp mRNA expression (**e**). (**f**) The FN-κB regulated IL-6 is expressed at protein by ELISA * *p* < 0.05; ** *p* < 0.005; *** *p* < 0.0005: Kruskal–Wallis test.

**Figure 3:**
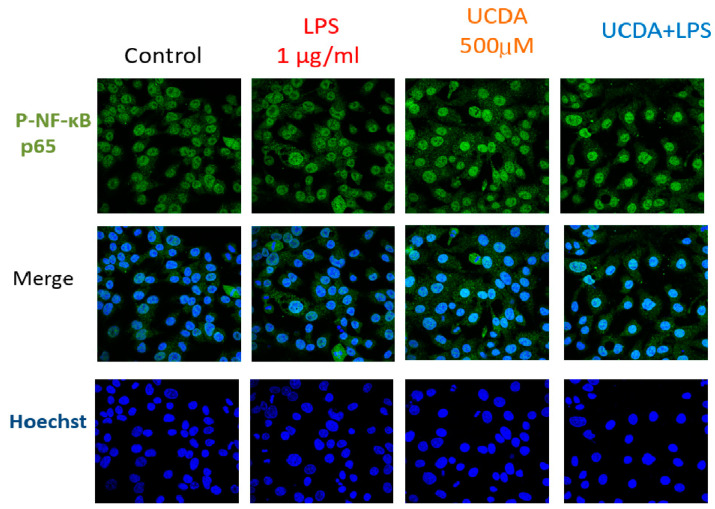
Effect of UDCA on the nuclear translocation of phospho-NF-κB p65 in LPS-induced HepG2 cells. Immunofluorescence assay and confocal microscopy image were applied to detect the distribution of phospho-NF-κB p65 in HepG2 cells treated with LPS alone or in combination with UDCA (LPS 1 µg/mL; ESO 25 µM)**.** The images were captured at 200× magnification.

**Figure 4:**
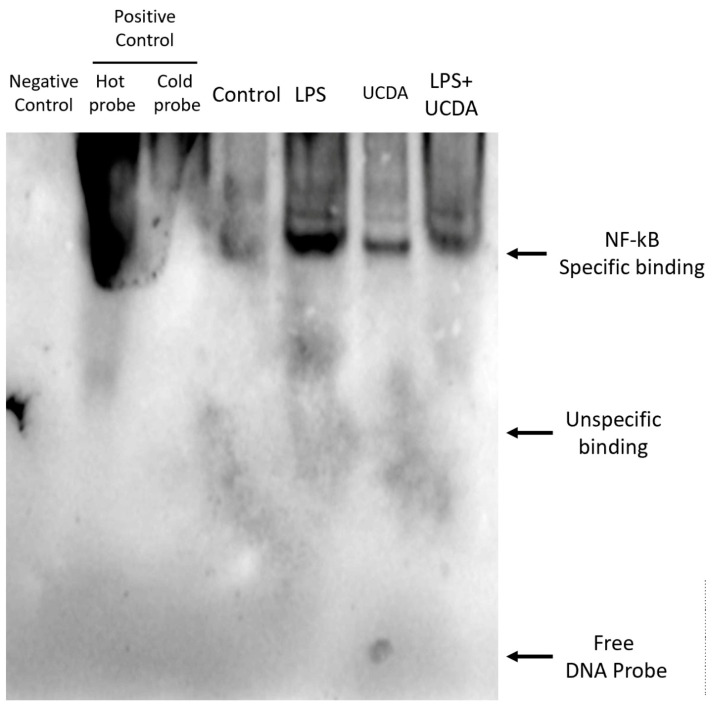
UDCA suppresses the NF-κB activity inside the nucleus of NF-κB in LPS-stimulated HepG2. Cells were treated with LPS (1 μg/mL) combined with/without UDCA (500 μM) for 24 h. EMSA was performed to determine the NF-κB activity in the nuclear fraction using a DNA probe specific to NF-κB.

Figure 1 shows that UDCA may have inhibitory effects on NF-κB activation induced by LPS. The results demonstrated that UDCA treatment inhibited p65 and p65 phosphorylation in HepG2 cells treated with LPS. Quantification of NF-κB and FXR expression is shown in Figure 1b. UDCA regulates LPS-induced NF-κB and FXR target genes and proteins, affecting TNF-α, IL-6, COX-2 production, and FXR target gene mRNA in HepG2 cells (Figure 2). A reduction in the binding activity of NF-κB to DNA indicated decreased NF-κB activation by confocal microscopy (Figure 3). This methodology allowed for a detailed examination of NF-κB expression patterns and subcellular localization, providing valuable insights into its role in inflammatory signaling pathways within HepG2 cells. UDCA also downregulated effects on NF-κB activation induced by LPS, as also demonstrated by EMSA (Figure 4).

### 3.2. UDCA on LPS-Induced FXR Expression

UDCA on Liver FXR expression in HepG2 cells, particularly under LPS induction, has produced significant and compelling findings (refer to Figure 1a,b). Our study focused on elucidating the dynamic relationship between UDCA treatment and FXR modulation in the context of LPS stimulation in HepG2 cells. Upon subjecting HepG2 cells to the combination of UDCA and LPS, we observed a marked downregulation of FXR expression. The notable suppression of FXR expression suggests a complex interplay between UDCA and the inflammatory environment induced by LPS.

### 3.3. UDCA Attenuating LPS-Induced TNF-α, IL-6, and COX-2 Expression

Figure 2 reveals a notable downregulation of LPS-induced TNF-α (Figure 2a), IL-6 (Figure 2b), and COX-2 (Figure 2c) expression following UDCA treatment. These findings are of considerable significance given the pivotal roles of IL-6, TNF-alpha, and COX-2 in the inflammatory response, particularly within hepatic contexts.

### 3.4. UDCA on the Interplay of SHP and CYP721 Expression

UDCA influences the expression of genes involved in bile acid synthesis and regulation within the liver. UDCA markedly decreases the expression of cyp7a1 mRNA, a key enzyme in the classical bile acid synthesis pathway. Concurrently, UDCA has been observed to significantly increase the expression of small heterodimer partner (shp) mRNA in the liver. These results reflect the known ability of FXR activation to effect bile acid via SHP-dependent and CYP7A-1-mediated bile acid synthesis effect (Figure 2d,e).

### 3.5. UDCA-Modulated Micro-RNA 21 Levels

**Figure 5:**
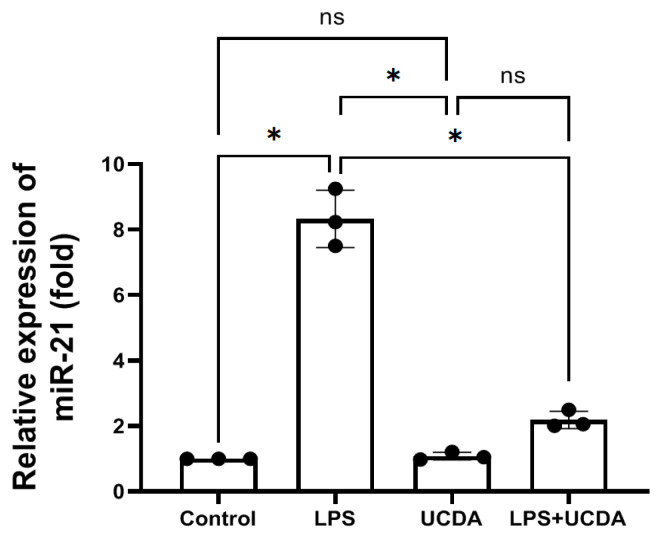
UDCA down-regulates miR-21 level induced by LPS in HepG2 cells. The miR-21 expression was determined by qRT-PCR. Each reaction was performed in triplicate, and mean values were used for calculating the relative fold change. Black dot is preseted as every experimental relative expression of miR-21. * *p* < 0.001 Kruskal–Wallis test. ns: no significance.

LPS treatment HepG2 cells upregulated the expression of micro-RNA 21. Figure 5 shows a notable downregulation of micro-RNA 21 levels following UDCA treatment in the context of LPS stimulation. UDCA itself did not induce or regress the expression of micro-RNA 21, but UDCA could mitigate the expression of LPS-induced micro-RNA 21 expression. This downregulation suggests a potential modulatory effect of UDCA on key regulatory pathways associated with hepatic inflammation and cellular response to LPS challenge.

## 4. Discussion

Our investigation established that the activation of FXR and NF-κB signaling induced by LPS contributes to the expression of associated cytokines and COX-2 in HepG2 cells. Co-treatment with UDCA effectively mitigates LPS-induced activation of FXR and NF-κB, the phosphorylation of NF-κB, as well as the expression of TNF-α, IL-6, and COX-2. These findings unveil the intricate mechanisms through which UDCA exerts its anti-inflammatory, and anti-carcinogenic effects, underscoring its clinical significance in the context of hepatic inflammation and carcinogenesis characterized by dysregulated cytokines and COX-2 expression. Furthermore, the impact of UDCA on LPS-induced changes in micro-RNA 21 expression provides valuable insights into its therapeutic potential for hepatic disorders. This study elucidates the interplay among UDCA, micro-RNA 21, and the FXR and NF-κB signaling pathways. UDCA was first characterized in the bile of the Chinese black bear and is physiologic hydrophilic dihydroxy bile acid [1,19]. UDCA has been found to increase bile flow and can change the hydrophobicity index of the bile acid pool. At present, UDCA is widely used and has been approved for cholesterol gallstone dissolution, as well as primary biliary cholangitis [7].

Experimental evidence suggests that UDCA operates through various mechanisms. It shields cholangiocytes from hydrophobic bile acid cytotoxicity by influencing mixed phospholipid-rich micelle composition, reducing bile acid cytotoxicity, and potentially lowering hydrophobic bile acid concentrations in cholangiocytes. Additionally, UDCA stimulates hepatobiliary secretion via p38MAPK and extracellular signal-regulated kinases, leading to the insertion of transporter molecules (e.g., bile salt export pump, BSEP, and conjugate export pump, MRP2) into the canalicular membrane of hepatocytes. Furthermore, UDCA protects hepatocytes from bile acid-induced apoptosis by inhibiting mitochondrial membrane permeability transition and potentially promoting a survival pathway [2].

NF-κB plays important roles in inflammation and carcinogenesis, including chronic liver diseases and tumorgenesis [24,25]. The precise mechanism by which UDCA attenuates LPS-induced NF-κB activation via the NF-Κb signaling pathway remains incompletely understood. It is hypothesized that UDCA may disrupt signaling pathways crucial for NF-κB activation, potentially by inhibiting IκB kinase or agents associated with NF-κB activation. IL-6 and TNF-α, influential pro-inflammatory cytokines, orchestrate a cascade of immune responses that can exacerbate inflammation and tissue damage when dysregulated. The observed reduction in their expression levels underscores the potential anti-inflammatory effects of UDCA. This effect is likely mediated through UDCA’s modulation of intracellular signaling pathways governing cytokine production and release. Previous reports have suggested that UDCA could inhibit NF-κB in microglia, and the esophagus could prevent hepatotoxicity [35,36,37]. UDCA could also downregulate several cytokines, including IL-8 and TNF-alpha [38]. In ethanol-induced hepatic bile acid accumulation and NF-κB activation in FXR-deficient mice, UDCA could attenuate NF-κB-medicated hepatic injury [39]. The inhibitory impact of UDCA on NF-κB activation, a central transcription factor in inflammatory signaling, further supports its role in alleviating the LPS-induced inflammatory milieu.

The FXR target gene, UDCA, has been shown to significantly influence the expression of genes involved in bile acid synthesis and regulation within the liver. Specifically, UDCA decreases the expression of cyp7a1 mRNA, a key enzyme in the classical bile acid synthesis pathway, when liver pathology is induced by lithocholic acid (LPD). This reduction in cyp7a1 mRNA expression can lead to a decrease in bile acid synthesis, potentially ameliorating conditions of cholestasis and reducing liver damage. Concurrently, UDCA has been observed to increase the expression of shp mRNA in the liver. SHP is a nuclear receptor that plays a crucial role in the negative feedback regulation of bile acid synthesis. By increasing SHP expression, UDCA contributes to the homeostatic control of bile acid levels, further protecting the liver from bile acid-mediated toxicity. This dual action of UDCA—decreasing cyp7a1 mRNA expression while increasing shp mRNA expression—illustrates UDCA on the FXR effect. Our results demonstrated that UDCA markedly decreases the expression of cyp7a1 mRNA and, concurrently, UDCA increases the expression of sshp mRNA in the liver. These results reflect the UDCA effect of FXR activation on bile acid via SHP-dependent and CYP7A-1-mediated bile acid synthesis effect.

The downregulation of COX-2, a pivotal enzyme in prostaglandin synthesis and a key player in inflammation, amplifies UDCA’s anti-inflammatory attributes. This inhibition of COX-2 expression holds particular relevance in hepatic pathologies, where excessive prostaglandin production contributes to liver injury and fibrosis. Additionally, considering the documented overexpression of COX-2 in cancers, UDCA’s potential anti-carcinogenic effect is noteworthy and warrants further exploration.

FXR, a key player in hepatic bile acid homeostasis and metabolic regulation, has recently gained attention for its involvement in inflammatory and carcinogenic pathways, particularly in metabolic-associated non-alcoholic fatty liver diseases. The observed downregulation of FXR expression hints at UDCA’s potential modulatory effect on this crucial receptor during liver inflammatory stress [39]. This effect is likely due to the intricate interplay between UDCA on FXR and NF-κB signaling pathways. Further mechanistic exploration of their roles in inflammation and carcinogenesis is warranted. These findings broaden our understanding of UDCA’s pharmacological properties and its potential implications in hepatic inflammatory and carcinogenic processes. The activation of the FXR has been demonstrated to exhibit anti-inflammatory properties. FXR activation is known to suppress the production of pro-inflammatory cytokines and chemokines induced by LPS, thereby presenting a potential avenue for mitigating the deleterious effects of LPS through modulation of the inflammatory response. According to the results of the present study, UDCA has a down-regulatory on FXR activity.

NF-κB and FXR are two distinct transcription factors that have important roles in regulating various cellular processes, including those involved in liver function and disease. NF-κB has its role in inflammation and immunity, as well as in liver disease and cancer. FXR also play a role in regulating bile acid metabolism and homeostasis, and its dysregulation has been implicated in liver disease and carcinogenesis. The interplay of NF-κB and FXR signaling has therapeutic potential, as evidenced by the effect of UDCA found in the current study. The relationship between these two factors in the liver has not been well investigated. A published study suggested that FXR activation can inhibit NF-κB signaling, potentially through the regulation of inflammatory cytokines and other mediators of the immune response [40]. Both NF-κB and FXR in the liver activated by LPS could apparently be downregulated by UDCA in the current results. The consequential downregulation of FXR expression under the influence of UDCA holds particular significance owing to the pivotal role of FXR in hepatocyte function and the broader regulation of metabolic processes. The influence of FXR extends to the modulation of bile acid synthesis, transport, and metabolism, as well as the regulation of lipid and glucose homeostasis. As such, the observed impact of UDCA on FXR expression implies potential implications for hepatic and metabolic functions. The regulatory interplay between UDCA and FXR offers a promising avenue for understanding and potentially manipulating anti-inflammatory responses and sheds light on the intricate molecular mechanisms that govern hepatic and metabolic regulation.

Our study elucidates the role of UDCA in modulating hepatic inflammation and carcinogenesis through the interactions with NF-κB and FXR signaling. The findings indicate that UDCA effectively downregulates the expression of pro-inflammatory markers such as COX-2, TNF-α, and IL-6. UDCA also reduces microRNA-21 levels, which are critical in the progression of hepatic inflammation and carcinogenesis. As the role of NF-κB signaling in inflammation, the downregulation of NF-κB signaling by UDCA has demonstrated its anti-inflammatory properties. NF-κB could be linked to chronic liver inflammation and carcinogenesis, including hepatitis, liver fibrosis, liver cirrhosis, and liver tumors. By attenuating NF-κB activation, UDCA potentially prevents deterioration of hepatic chronic inflammation, resulting in reducing the risk of liver cirrhosis and liver tumor. Furthermore, our results showed UDCA selective regulation of FXR signaling-related cyp7A1 and shp gene expressions. Thus, the effect of UDCA on FXR signaling could show the role of hepatic bile acid homeostasis and metabolism. In addition, the therapeutic potential of UDCA is further confirmed by UDCA’s impact on microRNA-21, a known regulator of multiple oncogenic processes. The significant decrease in microRNA-21 expression observed in our study suggests that UDCA may exert its anti-carcinogenic effects by modulating microRNA pathways.

To date, there are no reports in the literature on UCDA on MicroRNA-21, which features prominently in various pathological processes, encompassing inflammation, fibrosis, and tumorigenesis, particularly in hepatic contexts [33]. The ability of UDCA to modulate miR-21 levels suggests a potential intervention for hepatic inflammation and carcinogenesis marked by dysregulated miR-21 expression. This research paves the way for further investigations, including in vivo studies and clinical trials, to validate UDCA’s translational potential in mitigating hepatic inflammation and its consequences. The observed reduction in miR-21 expression may be attributed to UDCA’s multifaceted mechanisms of action. Combined with the downregulatory effects of UCDA on LPS-induced NF-κB signaling and FXR, the downregulation of miR-21 may consequently result in interactions among miR-21, NF-κB signaling and FXR, potentially leading to UCDA mitigating the inflammatory cascade initiated by LPS.

NF-κB plays a critical role in regulating the immune response to infection. It is involved in cellular responses to stimuli such as stress, cytokines, and bacterial or viral antigens. NF-κB also plays a key role in regulating the transcription of DNA, cytokine production, and cell survival. Apoptosis, or programmed cell death, is a process by which cells orderly die to remove cells that are no longer needed or are a threat to the organism, like infected or cancerous cells. The suppression of apoptosis by NF-κB could be considered a transcriptional event. In the context of diseases like hepatitis C, as illustrated in the provided abstract, NF-κB’s role becomes particularly significant [41]. The hepatitis C virus (HCV) NS5A protein modulates apoptosis in infected hepatocytes partly through the manipulation of NF-κB signaling pathways. By downregulating TLR4, a receptor known to activate NF-κB via MyD88-dependent pathways, NS5A effectively decreases NF-κB activation, leading to reduced expression of pro-apoptotic genes and increased survival of infected cells. The relationship between NF-κB and apoptosis is multifaceted and highly regulated, encompassing a balance between cell survival and death that is crucial for physiological processes, disease progression, and therapeutic potential. Further study for the effect of UDCA on NF-κB and apoptosis is worthy and indicates therapeutic potential.

## 5. Conclusions

Our investigation into the impact of UDCA on micro-RNA 21, FXR, and NF-κB signaling reveals that LPS-induced inflammation operates via FXR and NF-κB signaling in HepaG2 cells. Notably, UDCA effectively mitigated LPS-induced FXR, NF-κB, phosphorylation-NF-κB, TNF-α, IL-6, and COX-2 expression. This underscores UDCA’s anti-inflammatory properties, offering promise in hepatic disorders characterized by dysregulated cytokines and COX-2 expression. Furthermore, the influence of UDCA on LPS-induced changes in micro-RNA 21 expression provides pivotal insights into its therapeutic potential in addressing hepatic inflammation and carcinogenesis.

## Figures and Tables

**Table 1:** The forward and reverse sequences of the qPCR primers.

Primer Name	Forward	Reverse
IL-6	5′-TAGTCCTTCCTACCCCAATTTCC-3′	5′-TTGGTCCTTAGCCACTCCTTC-3′
TNF-α	5′-CAGGCGGTGCCTATGTCTC-3′	5′-CGATCACCCCGAAGTTCAGTAG-3′
COX-2	5′-GAATCATTCACCAGGCAAATTG-3′	5′-TCTGTACTGCGGGTGGAACA-3′
GAPDH	5′-GTCAAGGCCGAGAATGGGAA-3′	5′-CTCGTGGTTCACACCCATCA-3′

## Data Availability

Data are contained within the article.
